# Identifying structural connectivity priorities in eastern Paraguay’s fragmented Atlantic Forest

**DOI:** 10.1038/s41598-021-95516-3

**Published:** 2021-08-09

**Authors:** Noé U. de la Sancha, Sarah A. Boyle, Nancy E. McIntyre

**Affiliations:** 1 Department of Biological Science, Chicago State University, Chicago, IL USA; 2 Negaunee Integrative Research Center, The Field Museum of Natural History, Chicago, IL 60605 USA; 3 Department of Biology, Rhodes College, Memphis, TN USA; 4 Department of Biological Sciences, Texas Tech University, Lubbock, TX USA

**Keywords:** Conservation biology, Ecological modelling, Forest ecology, Forestry, Tropical ecology, Ecology

## Abstract

The Atlantic Forest of eastern Paraguay has experienced extensive recent deforestation. Less than one-third of the region is forested, and the remaining forest largely consists of isolated remnants with potentially disrupted connectivity for forest fauna. We used a graph theory approach to identify those forest remnants that are important in maintaining landscape structural connectivity for mammals in this fragmented forest. We quantified structural connectivity for forest remnants over the period 2000–2019 at three levels: the entire network of Atlantic Forest remnants in eastern Paraguay; at 10 smaller, nested spatial scales (40–10,000 m) encompassing a range of potential mammalian dispersal abilities; and at the level of individual remnants. We used 10 graph theory metrics to assess aspects of network complexity, dispersal-route efficiency, and individual remnant importance in supporting structural connectivity. We identified forest remnants that serve as important structural connectivity roles as stepping stones, hubs, or articulation points and that should be prioritized for connectivity conservation. Structural connectivity was constrained for organisms incapable of travelling at least 9–12 km (farthest distances between nearest-neighboring forest remnants depending on whether smaller remnants were included or not) and was particularly limited for area-sensitive forest-specialist mammals. With the increased forest loss and fragmentation that is occurring, the connectivity of this system will likely be further compromised, but most of the remnants that we identified as playing important roles for structural connectivity were outside of the country’s proposed “green corridor,” indicating additional areas where conservation action can be directed.

## Introduction

One of the hallmarks of the Anthropocene is fragmentation and resultant loss of habitat, with forests being one of the most impacted ecosystems^1^. Global forest area was reduced from 4128 million ha in 1990 to 3999 million ha in 2015^2^. Of the forest cover that remains, ~ 70% is within 1000 m of the forest’s edge, indicating pervasive fragmentation of formerly contiguous forests into small remnants^1^. These effects have been particularly noted within tropical forests^2–4^, where much of the remaining forest exists as small, isolated remnants that are under threat from future deforestation^5^. Continuation of current patterns of tropical forest fragmentation will result in a sharp increase in the number (and a decrease in the size) of forest remnants^6^. Effective tropical forest biodiversity conservation will only be possible if critical remnants can be identified for protection based not only on their size but also on their spatial positioning that combats isolation by promoting biotic flows through a fragmented habitat network.

One of the primary tropical forest ecosystems that has been subject to fragmentation is the Atlantic Forest (AF), second-largest forest in South America after the Amazon Forest. Ranging from eastern Paraguay into Argentina and extending to the Brazilian coastline as far north as Rio Grande do Norte ^7,8^, the AF is a global hotspot of biodiversity, with high species richness and endemism^9–11^. It has experienced extensive rates of deforestation^12–15^, and these patterns in deforestation continue^16–18^. This is thus a fragmented forest that is vulnerable to increasing isolation of remaining habitat areas^19^.

Paraguay has one of the highest rates of deforestation in the world^20,21^. The portion of the AF found in eastern Paraguay harbors a distinct assemblage of biota^8,22–25^. Forest fragmentation has occurred relatively recently and rapidly there^12,16,17,26–28^. In the early 1970s, approximately 73% of the Atlantic Forest ecoregion was forested^12^, but by 2013 less than one-third of the area was forested^17^. Recent efforts have started to document the biodiversity of this diminishing forest system^22,23,29–31^. The effects of forest fragmentation on this biodiversity are still mostly unknown. The few analyses about the effects of AF fragmentation in Paraguay that have been conducted have universally indicated that forest loss and isolation negatively affect the abundance and distribution of species^30,32–34^.

Given Paraguay’s unique yet poorly known biota as well as the country’s recent and rapid deforestation, information is urgently needed to identify which forest remnants are the most important in supporting regional biodiversity^30^. Such information can now be provided by techniques that are not predicated on species-specific data and can identify which forest remnants are located in areas that potentially link protected areas or large areas of relatively intact forest, thereby enhancing connectivity of a fragmented habitat network.

Landscape connectivity can be assessed in terms of structure or function: structural connectivity is how the spatial arrangement of the landscape potentially affects movement, whereas functional connectivity is a direct assessment of how organisms respond to landscape structure^35^. Structural connectivity is quantified in terms of the density and proximity of habitat patches and as such is relatively straightforward, not requiring species-specific movement data and offering a good tradeoff between effort and detail^36^. In contrast, functional connectivity is, by definition, species-specific and thus requires data that simply may not exist for many taxa. In the absence of such movement data, structural connectivity can be assessed over a range of distances that represent a range of species’ potential dispersal distances, allowing the results to then be used to make inferences about potential functional connectivity. A structural connectivity approach is an incomplete assessment of overall landscape connectivity, but it is better than no assessment (particularly given the rapid pace of forest and biodiversity loss), and it focuses on habitat patches, the actual conservation units being protected on the ground. Moreover, structural connectivity models serve as good null models against which functional connectivity assessments can be compared when/if species-specific data on land cover permeabilities become available. Therefore, quantifying and maintaining structural connectivity has been a primary focus of conservation activities^35^.

Given current rates of deforestation and habitat fragmentation worldwide, it is important to implement rigorous and rapid methods to assess landscape connectivity, particularly for sensitive taxa like forest mammals. Graph theoretical methods are one such approach that has been valuable in quantifying structural connectivity among habitat patches^36,37,38^. In this approach, an ecological network is described as a graph composed of nodes (forest remnants) and links (dispersal paths between remnants). This approach allows for identification of nodes that are crucial in supporting overall network cohesion; because identification of priority sites is a key goal of conservation, graph theory is an important rapid-assessment tool^37,39^. In this approach, the overall network of remnants can be quantified by metrics that assess node density, path redundancy, and network resilience^40,41^. In addition, the roles of individual forest remnants in facilitating connectivity through the network can be determined^42,38^.

This approach is not predicated on species-specific data (rather, it is a landscape-based approach that examines habitat patches). However, the connectivity patterns found can then be examined with respect to various species’ known dispersal distances. We focus here on forest mammals because as a group, mammals are sensitive to habitat fragmentation and habitat patch size^43–45^. However, our approach could be applied simultaneously to any taxa in the same forest remnants. Dispersal capacities of mammalian taxa vary widely in the AF, given the wide range of body sizes and home range sizes (Table 1), making this group a good model for effects of forest fragmentation on connectivity for biota in general. We therefore used a graph-theoretical approach to address the following questions: (1) What is the current pattern of forest cover within the AF of eastern Paraguay, and how has this pattern changed from 2000 to 2019? (2) How have patterns of structural connectivity within this forest changed over this time period as a result of fragmentation? (3) How does structural (and potential functional) connectivity of the network of forest remnants differ for species that differ in their vagility? (4) Which forest remnants in eastern Paraguay should be prioritized for conservation, based on their importance in maintaining structural (and potential functional) connectivity?

**Table 1:** Documented maximum movement distances (m) of mammals from the Atlantic Forest between forest remnants or otherwise into the surrounding non-forested matrix. A + indicates species that are not found in Paraguay sensu de la Sancha et al^24,62^ but potentially function similarly to closely related taxa in Paraguay.

Order	Genus	Species	Distance (m)	Technique
Carnivora	*Panthera*	*onca*	7500 m/day (6000–9300 m/day)	Telemetry^47^
Carnivora	*Panthera*	*onca*	7000–15,400 m/day	Telemetry^48^
Carnivora	*Leopardus*	*pardalis*	4000 MMDM	Camera Trapping^81^
Perissodactyla	*Tapirus*	*terrestris*	6112 Max	Telemetry^82^
Chiroptera	*Artibeus*	*fimbriatus*	3700	Mark/Recapture^83^
Chiroptera	*Artibeus*	*jamaicensis*	1200	Mark/Recapture^83^
Chiroptera	*Artibeus*	*lituratus*	4900	Mark/Recapture^83^
Chiroptera	*Carollia*	*perspicillata*	3700	Mark/Recapture^83^
Chiroptera	*Desmodus*	*rotundus*	1600	Mark/Recapture^83^
Didelphimorphia	*Caluromys*	*philander* +	90	Mark/Recapture^84^
Didelphimorphia	*Didelphis*	*aurita*	73.06	Spool^85^
Didelphimorphia	*Didelphis*	*aurita*	640	Mark/Recapture^86^
Didelphimorphia	*Gracilinunus*	*microtarsus* (males) +	84.60 ± 8.67	Mark/Recapture^87^
Didelphimorphia	*Gracilinunus*	*microtarsus* (males) +	59.89 ± 7.67	Mark/Recapture^87^
Didelphimorphia	*Marmosops*	*incanus* +	715	Mark/Recapture^86^
Didelphimorphia	*Marmosops*	*incanus* +	53.75 ± 3.15	Mark/Recapture^87^
Didelphimorphia	*Metachirus*	*nudicaudatus*	72.88	Spool^85^
Didelphimorphia	*Metachirus*	*nudicaudatus*	100	Mark/Recapture^54^
Didelphimorphia	*Micoureus*	*demerarae* +	850 (491–1097)	Telemetry^88^
Didelphimorphia	*Micoureus*	*demerarae* +	800	Mark/Recapture^54^
Didelphimorphia	*Micoureus*	*demerarae* +	300	Mark/Recapture^54^
Didelphimorphia	*Micoureus*	*paraguayanus*	375	Mark/Recapture^86^
Didelphimorphia	*Philander*	*frenauta*	794	Mark/Recapture^84^
Didelphimorphia	*Philander*	*frenauta* (females)	99.24 ± 49.93	Spool^89^
Didelphimorphia	*Philander*	*frenauta* (males)	126.44 ± 62.16	Spool^89^
Rodentia	*Akodon*	*cursor* +	335	Mark/Recapture^54^
Rodentia	*Akodon*	*montensis*	61.6 ± 5.9	Mark/Recapture^90^
Rodentia	*Akodon*	*montensis*	26.3 ± 1.37	Mark/Recapture^87^
Rodentia	*Delomys*	*sublineatus*	38.14 ± 3.12	Mark/Recapture^87^
Rodentia	*Delomys*	*sublineatus*	55.2 ± 6.7	Mark/Recapture^90^
Rodentia	*Nectomys*	*squamipes*	315	Mark/Recapture^54^
Rodentia	*Oligoryzomys*	*nigripes* (females)	23.39 ± 4.85	Mark/Recapture^87^
Rodentia	*Oligoryzomys*	*nigripes* (males)	32.95 ± 3.96	Mark/Recapture^87^
Rodentia	*Rhipidomys*	*mastacalis*	180	Mark/Recapture^86^
Rodentia	*Sooratomys*	*russatus* (females)	53.51 ± 19.23	Mark/Recapture^87^
Rodentia	*Sooratomys*	*russatus* (males)	51.71 ± 7.25	Mark/Recapture^87^
Rodentia	*Thaptomys*	*nigrita*	38.29 ± 5.65	Mark/Recapture^87^

## Results

### Forest loss and fragmentation

**Figure 1:**
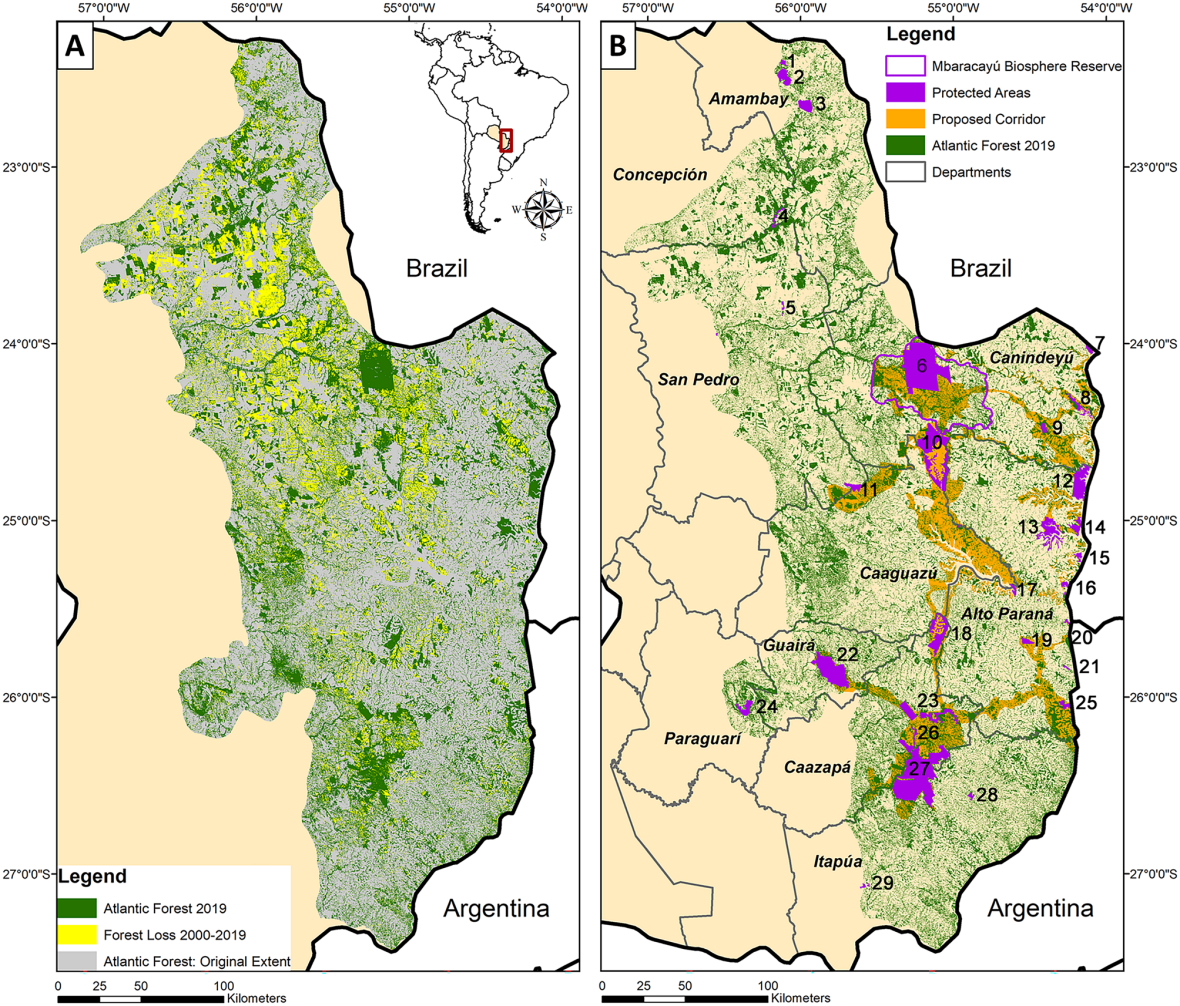
(**A**) Map of the original extent of the Atlantic Forest in Paraguay (gray; from Olson et al.^46^) with the extent of forest remaining in 2019 (green), and the amount of forest lost between 2000 and 2019 (yellow; Hansen et al.^21^). (**B**) Map of the 2019 Atlantic Forest in Paraguay, with currently recognized protected areas (UNEP-WCMC and IUCN 2020), proposed Paraguay Biodiversity Corridor (PBC, sensu Esquivel et al.^29^), and the 2019 forest cover. The protected areas are: (1) Estrella Reserve; (2) Arroyo Blanco Reserve; (3) Cerro Corá National Park; (4) Kaí Rague Private Nature Reserve; (5) Laguna Blanca Reserve; (6) Mbaracayú Forest Nature Reserve; (7) Mbaracayú Binational Nature Reserve; (8) Carapá Nature Reserve; (9) Itabó Ecological Reserve; (10) Morombí Nature Reserve; (11) Capiibary Ecological Reserve; (12) Limoy Nature Reserve; (13) Itabó Biological Reserve; (14) Yvyty Rókai Nature Reserve; (15) Pykyry Nature Reserve; (16) Tati Yupi Nature Reserve; (17) Yguazú Nature Reserve; (18) Ypeti Nature Reserve; (19) National Monument Kuri’y; (20) Maharashi Nature Reserve and Moises Bertoni Scientific Monument; (21) Tabucai Nature Reserve; (22) Ybytyruzu Management Reserve; (23) Caazapá National Park; (24) Ybycuí National Park; (25) Ñacunday National Park; (26) Tapyta Nature Reserve; (27) San Rafael National Park; (28) Edelira Managed Resource Reserve; (29) Cuenca del Arroyo Tacuary Chopy Sayju Nature Reserve. Maps were generated in ArcGIS 10.7^71^.

**Figure 2:**
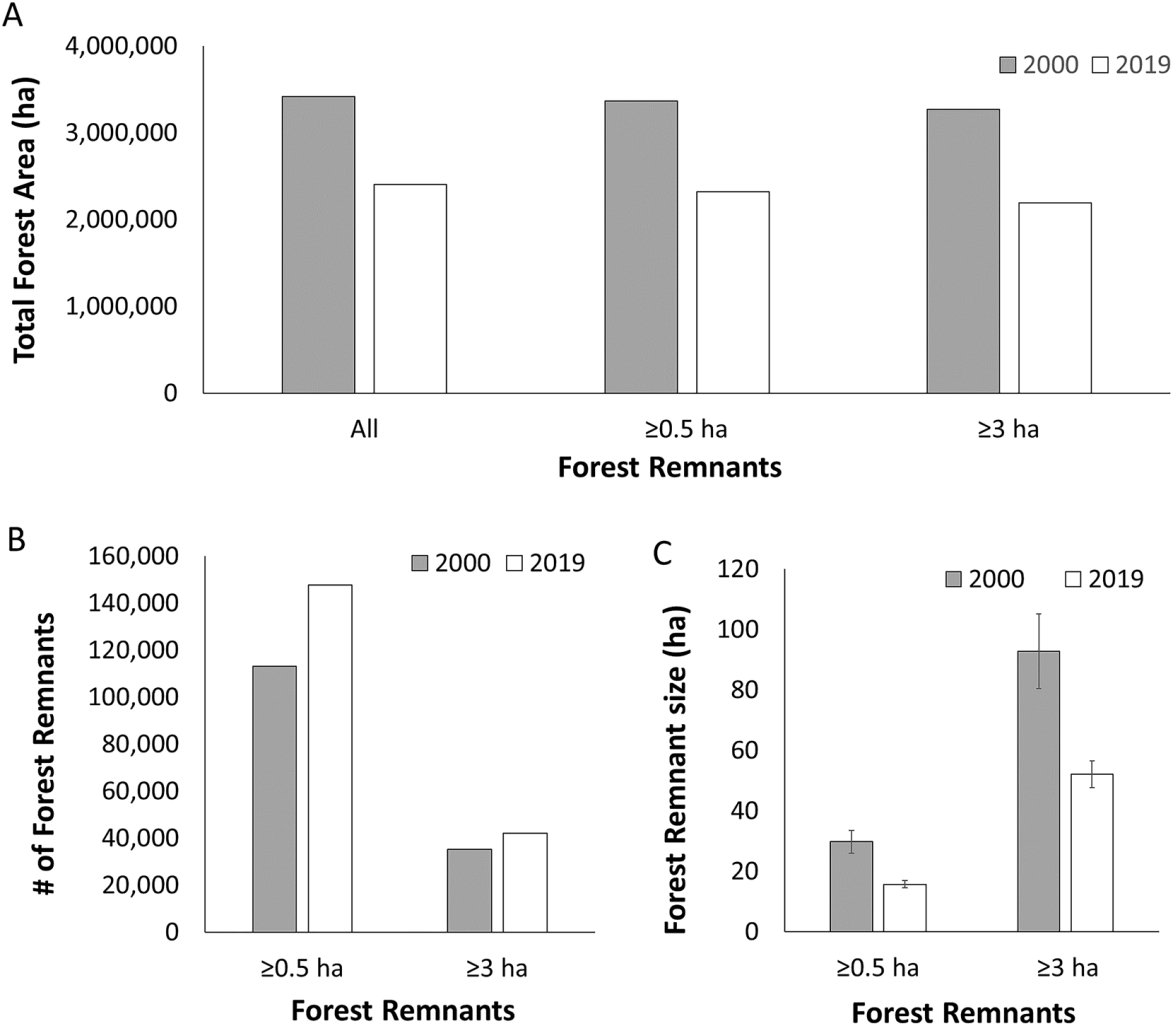
Interior Atlantic Forest in Paraguay (**A**) decreased by approximately 30% from 2000 to 2009, with (**B**) an increase in the number of forest remnants and a decrease in mean size during this time period. (**C**) Mean remnant size decreased from 2000 to 2019 when considering all forest remnants ≥ 0.5 ha as well as when considering all forest remnants ≥ 3 ha.

The Atlantic Forest in Paraguay has been heavily impacted in recent years. Using the extent of the Atlantic Forest ecoregion defined by Olson et al.^46^, we found that the geographic extent of this ecoregion in Paraguay was 45.3% forested in 2000, but by 2019 forest cover dropped to 31.8% of the ecoregion in Paraguay. Between 2000 and 2019, the total amount of Interior Atlantic Forest in Paraguay decreased by 29.7% of the forest cover that was present in 2000 (Figs. 1A, 2). When only forest remnants ≥ 0.5 ha and then ≥ 3.0 ha were considered, the decrease in forested area was 31.1% and 32.9%, respectively (Supplementary Table 1).

**Figure 3:**
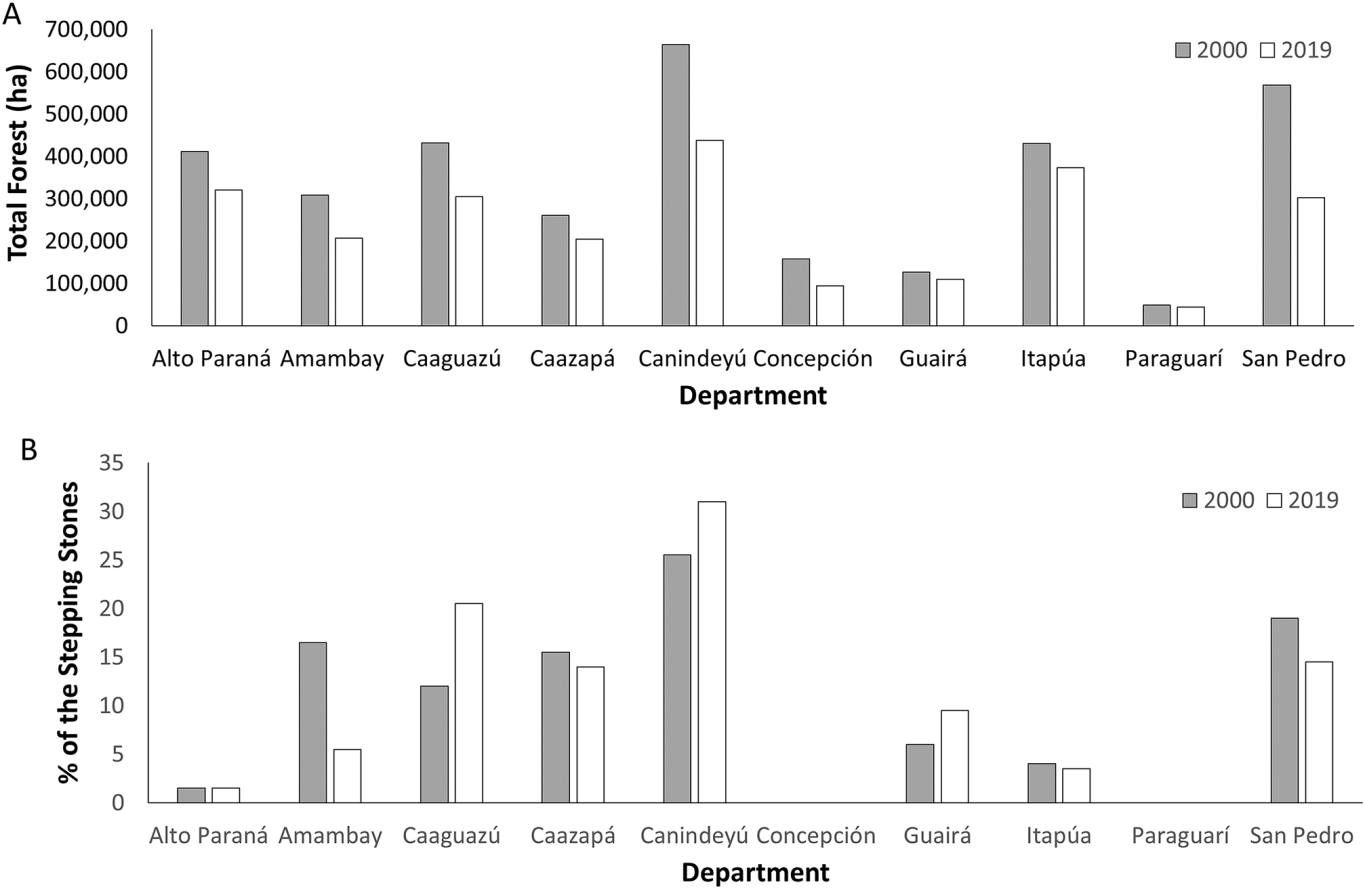
Forest loss between 2000 and 2019 occurred in (**A**) all departments, with Canindeyú and San Pedro having the greatest forest loss; while (**B**) of the top 200 stepping stones, most were located in Canindeyú, Caaguazú, and San Pedro.

The Atlantic Forest spans ten departments in Paraguay (Fig. 1). Canindeyú had the greatest percent of the Atlantic Forest within its departmental boundaries (19.4% and 18.1% in 2000 and 2019, respectively; Fig. 3). However, Canindeyú also experienced the third-greatest percent change between 2000 and 2019, with a reduction by 34.1% of the 2000 forest cover. San Pedro, which was ranked second in 2000 for the amount of Atlantic Forest within its department, experienced a reduction by 46.9% of the forest by 2019 (Supplementary Table 2).

Between 2000 and 2019, the number of forest remnants increased (remnants ≥ 0.5 ha: 30.6% increase; remnants ≥ 3 ha: 19.4% increase) and the mean remnant size decreased (remnants ≥ 0.5 ha: 47.2% decrease; remnants ≥ 3 ha: 43.9%; Fig. 2). This decline occurred throughout the region, further isolating designated protected areas (Fig. 1B). The total area of Atlantic Forest within the protected areas decreased by 20.5% between 2000 and 2019. Overall, the amount of remaining Atlantic Forest within protected areas was 11.8% in 2000 and 13.3% in 2019. However, when considering the number of individual forest remnants, fewer than 5% of the forest remnants were present within protected areas in 2000 (remnants ≥ 0.5 ha: 2.4%; remnants ≥ 3 ha: 2.1%) and 2019 (remnants ≥ 0.5 ha: 4.2%; remnants ≥ 3 ha: 3.9%).

### Structural connectivity—overall network metrics

There were fewer large than small forest remnants, with increasing complexity for these more fragmented networks (Figs. 1, 2, 3). Several metrics showed changes associated with remnant abundance and size (Fig. 3). For example, coalescence distance, number of nodes, and number of links were greater for the more fragmented network in 2019 than in 2000 (Table 2; each metric is defined in the Methods). Graph density, maximum cluster size, mean cluster size, modularity, and graph diameter increased with increased deforestation, whereas average nodal connectance and transitivity (clustering) decreased (Table 2).

**Table 2:** Overall-network connectivity metrics for networks of forest remnants in eastern Paraguay in 2000 and 2019.

Connectivity indices	2000	2019	Change	% Difference
Coalescence Distance (m)	6791	10,547	− 3756	55.3
Number of Nodes	35,232	42,081	− 6,849	19.4
Number of Links at Coalescence	1,361,630	4,012,771	− 2,651,141	194.7
Graph Density	0.00219	0.00453	− 0.00234	106.8
Average Nodal Connectance	31	18.8	12	− 39.4
Graph Diameter (m)	609,443.70	603,247.40	6,196.30	− 1.0
Transitivity	0.62732	0.62445	0.00287	− 0.5
Maximum Cluster Size	10,547	6791	3756	− 35.6
Mean Cluster Size	35,232	42,081	− 6849	19.4
Modularity	− 0.000034	− 0.000027	− 0.00001	− 20.6

**Figure 4:**
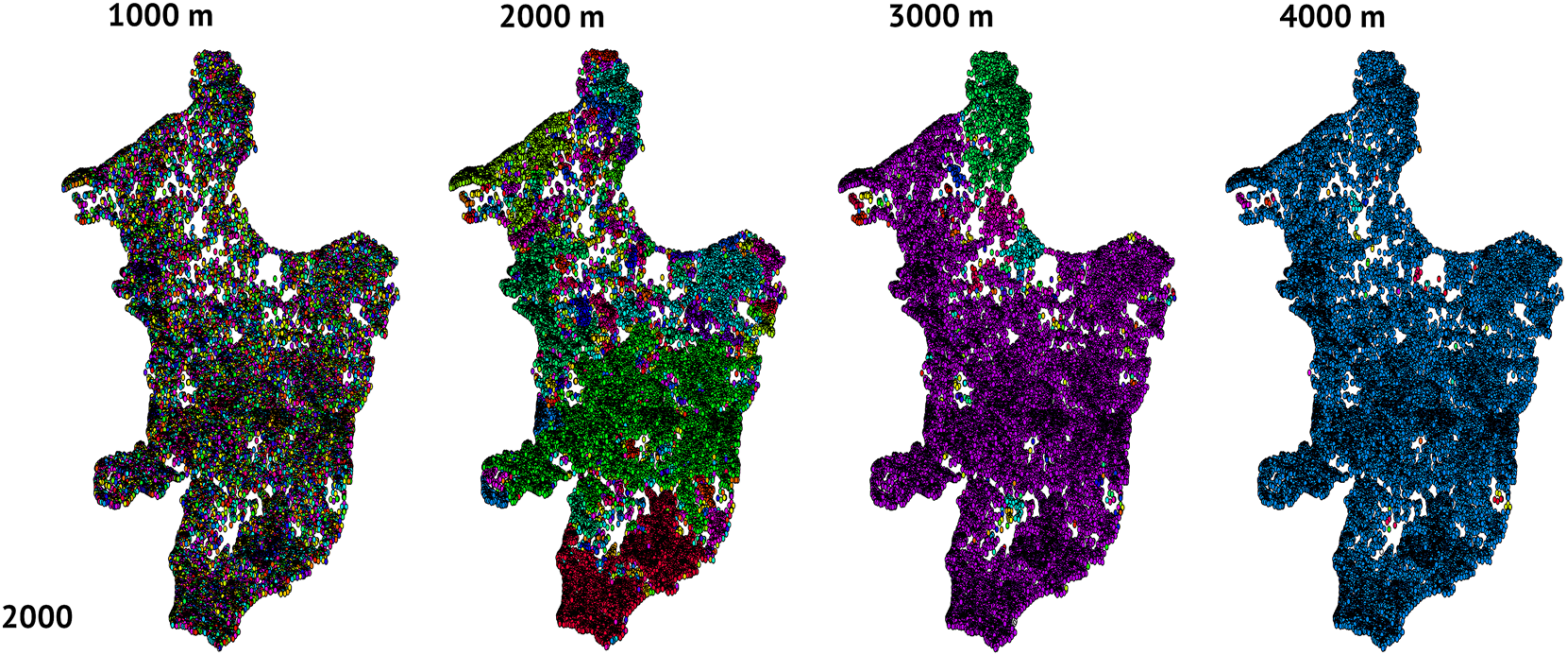
Comparison of global coalescence based on different dispersal distances (1000 m, 2000 m, 3000 m, 4000 m, 5000 m) comparing the patterns of clusters for 2000. Remnant centroids are shown as colored points. If two or more remnants are within the indicated dispersal distance from each other, they are denoted with the same randomly chosen color to indicate that they are part of the same cluster. Networks built with dispersal distances < 1000 m had too many isolated nodes that were too far apart to form clusters to discern any patterns. Figures were generated using RStudio.

**Figure 5:**
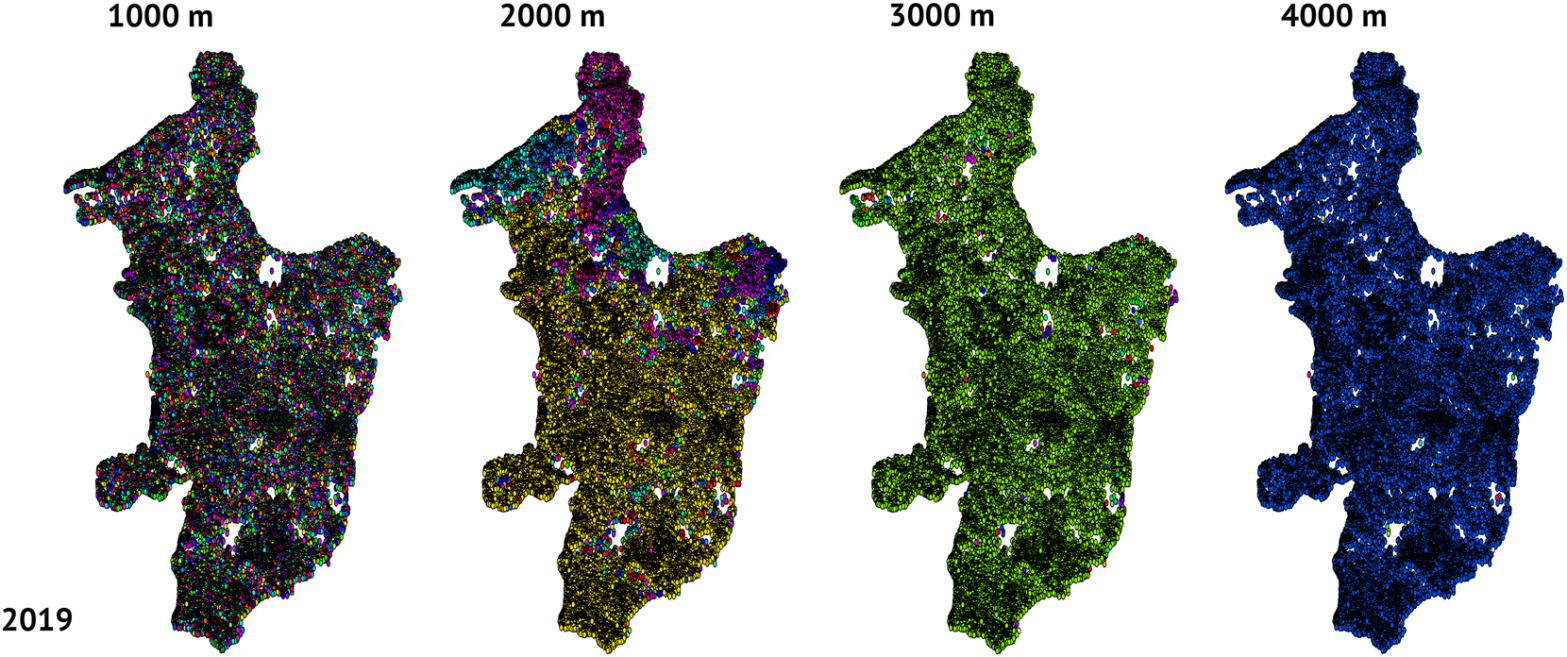
Comparison of global coalescence based on different dispersal distances (1000 m, 2000 m, 3000 m, 4000 m, 5000 m) comparing the patterns of clusters for 2019. Remnant centroids are shown as colored points. If two or more remnants are within the indicated dispersal distance from each other, they are denoted with the same randomly chosen color to indicate that they are part of the same cluster. Networks built with dispersal distances < 1000 m had too many isolated nodes that were too far apart to form clusters to discern any patterns. Figures were generated using RStudio.

At dispersal distances below the coalescence distance, clustering of forest remnants was apparent (Figs. 4, 5). Because ~ 80% of the maximum movement distances listed in Table 1 are below 1000 m, the few clusters that did form at distances < 1000 m may be of importance for conservation depending on the conservation status of less-mobile species). Additionally, differences with potential dispersal distance and year were evident: with increasing dispersal capacity (reading each row left to right in Figs. 4, 5), the network of remnants went from a collection of isolated remnants (many colors on the left) to a cohesive collection with increasing dispersal capability (single color by 4000 m on the right). Similarly, comparing across years for the same dispersal distance (reading each column top to bottom in Figs. 4, 5) indicates differences between years with the creation of more fragments in 2019 compared to 2000 (the denser plots in the bottom row).

**Figure 6:**
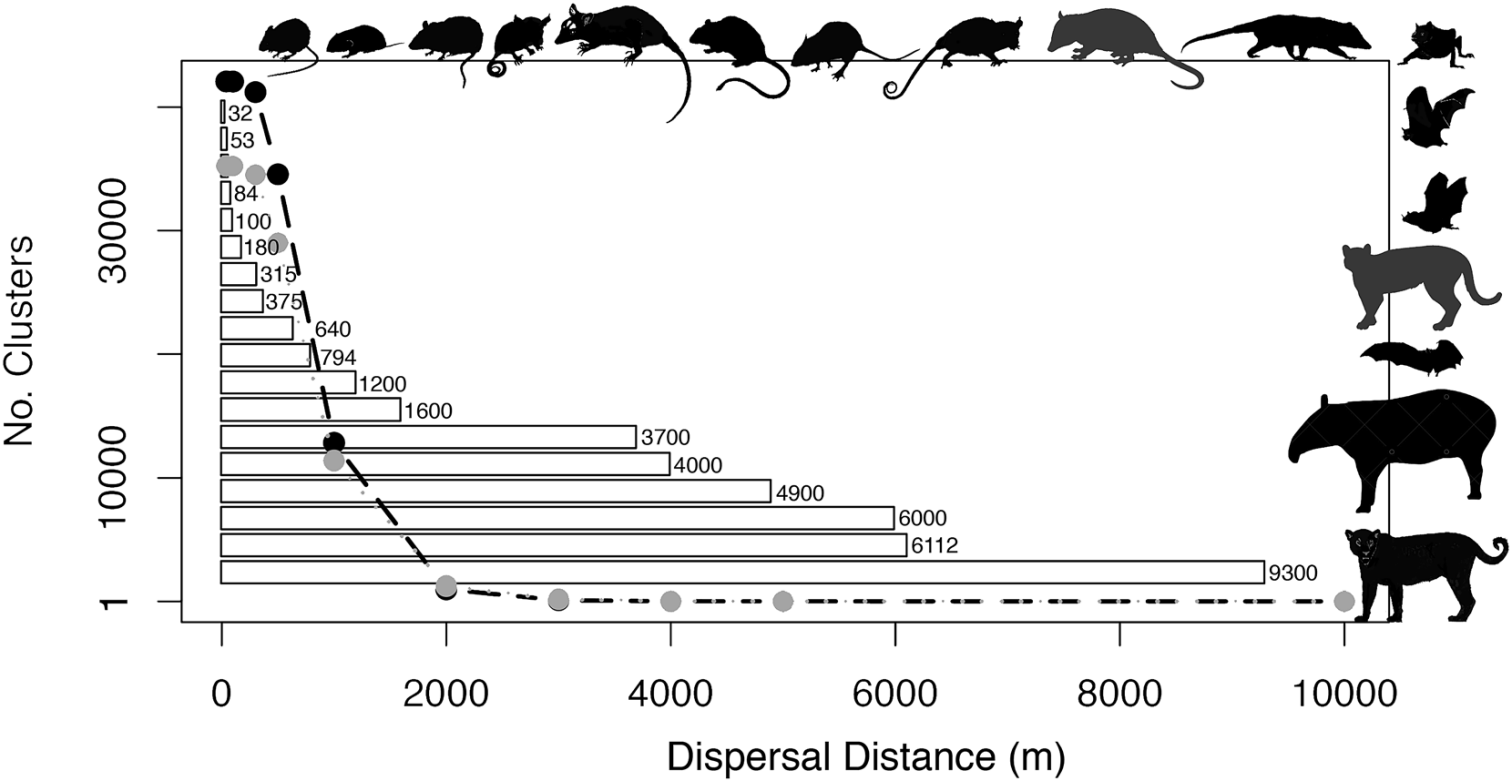
Comparison of number of clusters by distance for 2000 (gray line) and 2019 (black line). Superimposed bars represent documented field travel distances (m) for selected Atlantic Forest species. From top from left to right: Oligoryzomys nigripes, Thaptomys nigrita, Akodon montensis, Gracilinunus microtarsus*, Metachirus nudicaudatus, Rhipidomys mastacalis*, Nectomys squamipes, Marmosa paraguayana, Didelphis aurita, Philander frenata, on right margin top to bottom, Desmodus rotundus, Artibeus fimbriatus, Carollia perspicillata, Leopardus pardalis, Artibeus lituratus, Tapirus terrestris, and *Panthera onca* (see Table 1 for entire details and additional species). *Identifies species not found in Paraguay but which have generic counterparts that might have similar dispersal abilities. Species silhouettes were generated with Photoshop and figures were generated in RStudio and compiled using BioRender.com.

The numbers of clusters decreased with increasing potential dispersal distance independent of the size of forest remnants being considered (Figs. 4, 5, 6; see details and additional summary statistics in Supplementary Tables 3 and 4); for example, in 2000 there were 35,232 clusters at a dispersal distance of 40 m but only 1 cluster at a distance of 10,000 m, and for 2019 there were 42,079 clusters at 40 m and 2 at 10,000 m (Figs. 4, 5). Given the large number of clusters, at the regional scale, connectivity maps constructed at distances below 1000 m seem to show little to no structure (Figs. 4, 5). Other connectivity metrics similarly varied as a function of dispersal distance and remnant size per year (Figs. 4, 5).

### Structural connectivity—node-level metrics

**Figure 7:**
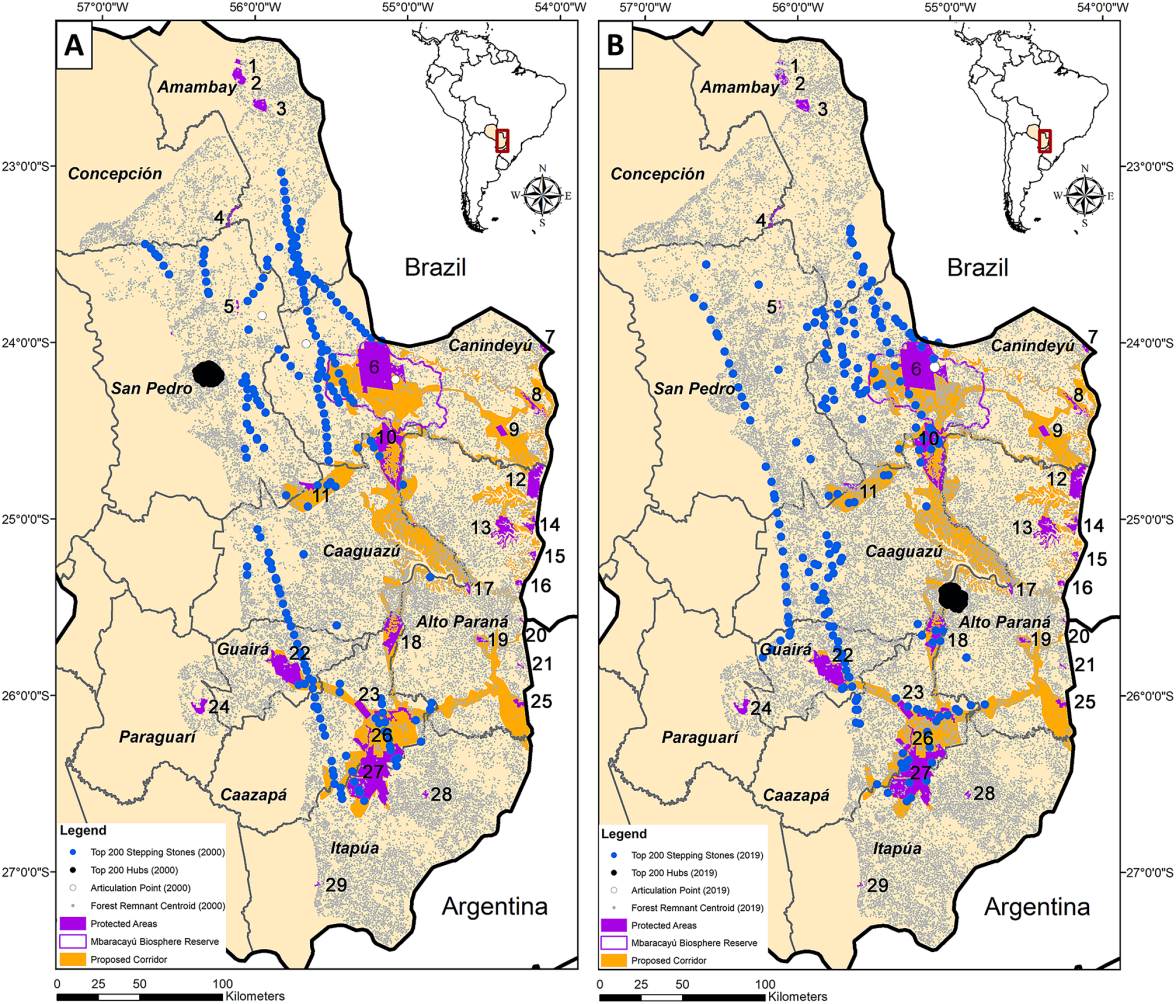
Maps of stepping stones (blue), articulation points (white), and hubs (black) for the network of forest remnants ≥ 3 ha (gray), currently recognized protected areas (UNEP-WCMC and IUCN 2020), proposed Paraguay Biodiversity Corridor (PBC, sensu Esquivel et al.^29^) in (A) 2000 (n = 35,232 forest remnants ≥ 3 ha) and (B) 2019 (n = 42,081 forest remnants). Forest remnants are denoted by their centroid. The protected areas are identified in the Fig. 1 legend. Maps were generated using ArcGIS 10.7^71^.

Each node-level metric we used is defined in the Methods. The identities of the 200 top-ranked stepping stones and top 200 hubs in 2000 (Fig. 7A) and 2019 (Fig. 7B) were in many ways consistent between the networks. However, between 2000 and 2019 there was a considerable shift in the top-200 hubs eastward from the department of San Pedro to Alto Paraná (Fig. 7). This shift mirrors the pattern of deforestation between 2000 and 2019, with regions of forest loss in the northern and western regions of the Atlantic Forest in Paraguay (Fig. 1A). Of the top-200 stepping stones, most were found in the departments of Canindeyú, Caaguazú, and San Pedro (Fig. 3), specifically in the western regions of these three departments (Fig. 7). The majority of stepping stones were not within protected areas (e.g. Figures 1B and 7B for 2019), and all three departments experienced substantial forest loss between 2000 and 2019 (a loss of 29.3–46.9% of the 2000 forest extent in these departments; Fig. 3). The presence of a single articulation point in 2019 indicates a vulnerable portion of the habitat network (Fig. 7B).

## Discussion

We documented current patterns of structural connectivity in the AF of eastern Paraguay, quantified how this pattern changed from 2000 to 2019, and identified areas where connectivity could be enhanced. For example, comparisons between 2000 and 2019 showed that the vast majority of stepping stones were located in roughly the same portions of the country (Figs. 3, 6), and with changing fragmentation the placement of the those stepping stones fluctuated, largely in regions with increased deforestation (Figs. 1 and 6). Similarly, there were three articulation points in 2000, but only one of those remained in 2019 (Fig. 7). Finally, there was a dramatic shift in hubs from central San Pedro Department in 2000 down to western Alto Paraná in 2019. These differences between 2000 and 2019 indicate that connectivity is not a static property of landscapes and that conservation efforts should be based on areas consistently identified as important. For conservation planning, the most robust strategy would be to embrace connectivity dynamics and preserve networks that are resilient against potential future changes.

Node-level assessments allowed us to identify consistent areas to prioritize for conservation. For example, we found an articulation point and a group of stepping stones between southern Amambay, western Canindeyú, into northern Caaguazú Departments (Fig. 7), in a region proposed as a biological corridor by Da Ponte et al.^17^. We also found consistent stepping stones from northern Department of Caazapá stretching north into the Department of Guirá, around Ybyturuzu Reserve, continuing north along western Department of Caazapá, and continuing into Department of San Pedro. Finally, we found a series of stepping stones around Reserva San Rafael in the Departments of Caazapá and northern Itapúa in both years, indicating that these are consistently important areas for structural connectivity of the forest network.

Thus, based on our most recent connectivity analyses, we recommend the following five regions that should be high priorities for connectivity conservation in eastern Paraguay: (1) the region of stepping stones along the southern rim of the Mbaracayú Forest Nature Reserve (Fig. 7B, point 6) that may promote connectivity with Morombí Nature Reserve (Fig. 7B, point 10); (2) the area to the northwest of Mbaracayú with stepping stones that extended to a series of medium-sized remnants near Kaí Rague Private Nature Reserve (Fig. 7B, point 4); (3) the region extending from Ybytyruzu Management Reserve (Fig. 7B, point 22) to a series of remnants in central Caaguazú, which could potentially continue to Ecological Reserve Capiibary (Fig. 7B, point 11); (4) the area extending from San Rafael National Park (Fig. 7B, point 27) to Ypeti Nature Reserve (Fig. 7B, point 18) and Ybytyruru Management Reserve (Fig. 7B, point 22); and (5) the most important hubs in 2019 formed a cluster between Yguazú Nature Reserve and Alto Paraná (Fig. 7B, points 17–18), denoting an area with a high density of forest remnants that could bridge these protected areas. There were three articulation points in 2000, only one of which remained in 2019 and was part of the Mbaracayú Forest Nature Reserve, so this important node is already within a protected area (Fig. 7). These protected areas are important aspects of the remaining forested landscape, but with less than 15% of the Atlantic Forest in Paraguay located within a protected area, conservation of the forest remnants outside of the protected areas is crucial for maintaining connectivity.

Our approach focused on structural connectivity manifested at three scales: the global network of Atlantic Forest remnants in eastern Paraguay, clusters of remnants at smaller dispersal distances, and individual remnant-level analyses. The global connectivity metrics allowed us to quantify the degree of fragmentation of the AF in eastern Paraguay as a whole. Examining a suite of smaller potential dispersal distances from 40 to 10,000 m allowed us to compare fragmentation experienced by animals with different vagilities; by examining habitat networks composed of remnants of increasing sizes, we were able to compare the levels of isolation experienced by animals with different area requirements. Finally, use of node-level metrics allowed us to identify five regions that should be deemed high-priority areas for supporting connectivity in the AF of eastern Paraguay. These results could then be examined with respect to regional mammals.

Our structural connectivity analyses indicate that the AF of eastern Paraguay is currently fragmented beyond distances that most non-volant mammals can travel (Table 1, Fig. 7). The implications of this finding is that there are species for which connectivity has been severed, others that can still possibly traverse the network, and still others that are too poorly known to determine how they are affected. For example, jaguars in the Paraguayan Atlantic Forest have been documented to travel as much as 9300 m^47^ and some individuals in the Brazilian Atlantic Forest have been documented as travelling more than 15,000 m^48^, which means that they could possibly traverse a fragmented forest network. However, these large felids are highly susceptible to poaching or hunting near forest edges^47^. Similarly vagile species (e.g. various bat species) may yet perceive the Paraguayan Atlantic Forest network as more cohesive, given their dispersal capacity (Table 1). Indeed, a recent evaluation of gene flow of *Artibeus lituratus* (a fairly large and robust species with large known dispersal distances; Table 1) throughout the Atlantic Forest of Paraguay showed no genetic differentiation with continuous forests in neighboring Argentina^34^. Furthermore, simulations based on genetic structure for this species estimate dispersal distances as high as 30 km^34^, well beyond our estimated coalescence distances, indicating that this species is likely able to navigate the fragmented forest network. Other bats with lower dispersal distance might also be able to disperse through the network via stepping stones. However, for most bats their documented dispersal distances are below the coalescence distances, meaning that they may exist in isolated patches.

The fragmented AF represents a very dense and complex habitat network, with many potential travel routes through it for those organisms capable of traveling at least 10,500 m (2019) in the non-forested inter-remnant landscape matrix, which is composed mainly of agricultural fields and livestock pastures. There are many factors that determine the ability of species to move in the matrix between remnants^49,50^. Megafauna like tapirs potentially have large dispersal distances (Table 1), but this is a highly threatened species that is regularly poached and hunted for subsistence where they are found in Paraguay^51^. Large opossums such as *Philander frenata*, *Didelphis aurita,* and *Metachirus nudicaudatus* adapt well to disturbed habitats and human-dominated landscapes, and are regularly found in the matrix surrounding forest patches^52–54^, whereas forest specialists like *Caluromys lanatus* might be more affected. Dispersal data are lacking for many rodents and small marsupial species, but the species for which such data are known (Table 1) tend to be species that occur in non-forested areas and even agroecosystems^55–57^. It is clear that the type of matrix affects dispersal through it in a strongly species-specific manner^49,53^. Thus we are not sure how forest-dependent species might adapt to fragmentation, although smaller forest remnants do show considerable loss of small mammal species^58^ relative to the largest remnants found in the region^22^. Even though there were numerous forest remnants present, the distances between them may be beyond the dispersal capacity of many animals. Finally, the presence of a single articulation point indicates that the AF in Paraguay is extremely fragile; losing this articulation point would further fragment the system, so this remnant should be a conservation priority.

When forest remnants of increasing sizes were examined (representing different thresholds of the amount of habitat needed to support area-sensitive species), varying patterns of connectivity were revealed. There were few stepping stones between some of the largest forest remnants left in eastern Paraguay (Fig. 7). These stepping stones are crucial in maintaining connectivity, and their locations were consistent across networks of forest remnants of different sizes. Thus, our analyses suggest that the largest forest remnants of this portion of the country are effectively isolated for most taxa. Although a metacommunity analysis along the entire Atlantic Forest from northeastern Brazil to Paraguay found that some species (e.g. *Didelphis aurita, Metachirus nudicaudatus, Marmosa demerarae*) can be found in forest remnants of various sizes^23^, many remnants may be too small to support populations. But even the smallest forest remnants may play valuable roles as stepping stones^59^ (Fig. 1). Similarly, the 200 top-ranked hubs were consistently located in a geographically restricted portion of southeastern Paraguay, isolated from other forest patches (Fig. 7).

A structural approach is not always a good measure of actual connectivity because it focuses on habitat patches and not organisms per se. For example, we measured the Euclidean distances between forest remnant centroids rather than their edges; this approach is computationally efficient and most appropriate when the distances between patches exceed the sizes of the patches themselves^60,61^. However, this approach will overestimate true dispersal distances between remnants. In addition, our structural analyses did not examine the functional responses of animals to land cover within the non-forested landscape matrix. In Paraguay, the vast majority of deforestation has been conducted to create livestock pastures and for soy cultivation^12^; these agroecosystems could potentially allow for dispersal between forest remnants for some taxa^55–57^. However, many of the species that are found in agroecosystems tend to be generalists and not endemics or forest specialists^55,56^. Information on the ability of forest-specialist mammals to travel in a non-forested matrix is limited. There is some information for a few taxa from the Brazilian AF, but comparatively little is known from Paraguay (Table 1). Such information is urgently needed for more comprehensive landscape conservation.

It was not surprising that large forest remnants were effectively isolated from each other for taxa with low vagility because of the sparse distribution of such remnants. Consequently, area-sensitive and low-vagility species are the ones that are most likely to be affected by AF fragmentation in eastern Paraguay. Current patterns of fragmentation will most strongly affect species with limited dispersal abilities, such as small or medium-sized non-volant mammals, and should probably disproportionately affect forest specialists. These effects are corroborated visually with our maps. The current range of inter-remnant distances is beyond the dispersal distance of many species (Fig. 7). For the most travel-restricted taxa (i.e., those incapable of traveling more than 1000 m), connectivity is strictly curtailed (Figs. 4, 5). At a distance of 2000 m, however, some habitat clustering emerges. At 3000 to 4000 m, the central portion of the Paraguayan AF becomes more connected. Beyond 10,000 m, clusters of remnants begin to coalesce into regional habitat areas.

## Conclusions

The AF of Paraguay has experienced an alarming rate of forest loss in the last few decades; given that the fauna of Paraguay are still so poorly known, effects of this deforestation are not yet fully appreciated^24,62^ and there is an urgent need to improve connectivity between forest preserves^47^. Our results indicate that fragmentation will disproportionately affect those species that cannot disperse farther than 9500 m; for area-sensitive species, those that cannot move at least 11,300 m within a non-forested matrix will be negatively affected. These distances are beyond the known movement distances for 24 regional forest mammals (Table 1).

Isolation is a strong predictor of biological diversity in fragmented landscapes^63,64^, and connectivity is a supporting driver of species diversity in forest fragments. The Paraguay Biodiversity Corridor initiative is part of the project Paraguay Biodiversidad, a collaboration with Itaipu Binacional and the World Bank, with an objective of conserving biological diversity of global importance while promoting the sustainable land use in the productive area of the Bosque Atlántico del Alto Paraná and associated ecosystems in Paraguay^65^. This initiative proposed a corridor system for eastern Paraguay^29^ in part informed by prior assessments for the larger Upper Parana AF Corridor network^66^. Based on the most current dataset from 2019, our results independently corroborate siting of some portions of this proposed green corridor and other proposed corridors in eastern Paraguay^17,29^ (e.g. some stepping stones and hubs in 2019 are in/near the proposed corridor) and also highlight many regions outside of the proposed corridor that may facilitate connectivity. For example, we identified a region of stepping stones that could potentially link San Rafael and Parque Nacional Caazapá (Fig. 7B, point 27) and Reserva Recursos Manejadas Yvytyrusu (Fig. 7B, point 22). Our analysis also consistently recovered stepping stones north of Yvytyrusu (Fig. 7B, point 22) towards Capiibary Ecological Reserve (Fig. 7B, point 11) or Carla María Farm, thereby highlighting a series of potentially valuable remnants that may not be currently protected but that could increase connectivity between two core areas as proposed by the green corridor^29^. Although our study was not meant to be a direct comparison to theirs because of vastly different objectives and approaches, it is interesting to note that our structural connectivity analyses largely reinforce what Esquivel et al.^29^ proposed for a different taxon (birds).

Strategic reforestation would help increase the connectivity of the AF by increasing node density and decreasing network coalescence distance; reforestation efforts that create stepping stone patches could be particularly fruitful. Reforestation efforts between Mbaracayú and Morombi (Fig. 7B, points 6 and 10), for example, could help link some protected areas, such as Parque Nacional Laguna Blanca or Reserva Natural Privada Kaí Rague (Fig. 7B, points 5 and 4). Additional candidate areas would extend northward from there toward Parque Nacional Cerro Cora (Fig. 7B, point 3). There were few stepping stones to facilitate connectivity between Morombi (Fig. 7B, point 10) south to San Rafael (Fig. 7B, point 27), indicating a need for investment to improve connectivity between these regions that are proposed as part of a nationwide green corridor system^29^. This strategy should be carefully considered, however, because it may have unintended negative consequences (e.g. negative edge effects or facilitating dispersal of invasive species^67,68^.

Surprisingly, we did not find many current stepping stones along the proposed green corridor network (Fig. 1) especially into Alto Paraná or western Canindeyú and western Caaguazú departments. Therefore, our recommendations for conservation actions are: use reforestation to restore connectivity between core areas, consider sites outside the green corridor network; strengthen conservation protections and minimize increased deforestation; and support research on Paraguay’s under-studied biota.

Conducting these sorts of connectivity analyses on numerous spatially explicit patches at a large spatial extent is still largely at the mercy of computational efficiency, but computational innovation is changing the kinds of analyses that can be performed. Additionally, high-resolution imagery can improve the identification of small remnants^69^. Our analyses were not feasible just a decade or so ago, and in another decade, we may well be able to examine most of South America on a tablet computer. Unfortunately, deforestation is occurring at a rate that is outpacing our ability to quantify its effects. Given the rates of deforestation worldwide^1^ and subsequent biotic impacts^55^, it is important and timely to identify priority sites for conservation. Many parts of the tropics are data-deficient and/or data-limited, which hampers conservation action. A graph-theoretic approach is one efficient means of identifying areas for targeted management.

## Materials and methods

### Forest data

We downloaded the Global Forest Change 2000–2019 dataset^21^ for the area representing the Atlantic Forest^46^ in Paraguay. The Hansen et al.^21^ data are updated annually and have been cited more than 6000 times. We downloaded forest cover data and forest-loss data (2000–2019) to calculate the extent of forest cover in 2000 and in 2019. The spatial resolution of the data was 30 m × 30 m, and we defined a pixel as forested if the estimated forest cover value by Hansen et al.^21^ was ≥ 50%. We converted these raster data to vector format, creating individual polygons (defined as a set of contiguous pixels by an 8-neighbor rule^70^) for each forest remnant. We then determined the area of every forest remnant in eastern Paraguay but focused the connectivity analyses on only those remnants that were at least 3 ha in area (N = 35,323 in 2000; N = 42,081 in 2019), due to limits in computation. We calculated the percent of forest loss from 2000 to 2019 and the extent to which the number of forest remnants increased and mean remnant size decreased. All forest analyses and maps were generated in ArcGIS 10.7^71^.

### Structural connectivity—overall network metrics

We implemented a graph theory based framework which has been shown to be a robust approach to assessing connectivity in fragmented landscapes^38^. We calculated the centroid coordinates for each forest remnant ≥ 3 ha. We modified the R script from Drake et al.^68^, using the package *igraph*^72^ in R 3.1.3^73^ to calculate structural connectivity metrics at three scales: for the network of forest remnants ≥ 3 ha as a whole, for clusters of remnants connected at smaller dispersal distances, and at the level of individual forest remnants for both 2000 and 2019. For the network as a whole, we first determined the coalescence distance, which is the farthest distance between nearest neighbors^68^; this metric determines at what dispersal distance the spatially separate forest remnants function as a single unit where organisms can disperse among them. At this distance, we calculated the number of links present (i.e., number of potential paths among remnants) and graph density, which is the unitless ratio of remnants linked within the coalescence distance to all remnants within the network (with higher values indicating greater path redundancy)^72^. We calculated average nodal connectance (average number of connections that each node has with other nodes, with lower values indicating remnants that are more isolated), graph diameter (longest most-direct path between the farthest two connected nodes, indicating the distance of the most efficient dispersal route through the network, moving from remnant to remnant), and transitivity (a unitless clustering coefficient that ranges from 0 to 1, with higher values indicating that most remnants are within the coalescence distance of at least two other remnants). See Csardi and Nepusz^72^ for more information on how each of these metrics is calculated and McIntyre et al.^41^ for ecological interpretation of these metrics. We were able to run the analyses for remnants ≥ 3 ha using package *igraph*^72^ in RStudio on a Microsoft Azure virtual machine that was optimized to memory size of 20 vCPUs, 160 Gb RAM, 32 data disks, 32,000 Max IOPS, and 750 Gb temporary storage. By not including remnants smaller than 3 ha due to computational limitations, we are underestimating overall landscape connectivity (effectively excluding the smallest species).

Although some bat species or megafauna might have the capacity to traverse most of the fragmented forest network, most mammals lack the vagility to be able to do so and thus would experience only local aspects of the network. Therefore, we also examined localized clusters of forest remnants within limited movement distances. To determine at what distance separate remnants would be considered part of the same cluster, we reviewed the literature on mammalian species and genera found in Paraguay^24,31^ or on closely related species of similar ecological function found in other parts of the AF^31,74^ to estimate distances traveled in non-forested areas surrounding forest remnants. We identified 24 species for which our localized structural connectivity analyses would be relevant (Table 1). While not all species are found in Paraguay, they are Atlantic Forest species with closely related taxa in the same genera^24^ and many have potentially ecologically equivalent species based on functional diversity patterns across this entire forest system^31^. We calculated the number of forest remnants clustered within a circle of increasing radius (40 m, 100 m, 300 m, 1000 m, 2000 m, 3000 m, 4000 m, 5000 m, and 10,000 m, which were round numbers comparable to averages found in the literature of maximum non-forested distances crossed by various taxa; Table 1). At each of these localized scales, we calculated the number of forest-remnant clusters, mean and maximum cluster size, graph diameter, and network modularity. The number of clusters represents the number of forest remnants within each of the distances 40–10,000 m, the mean and maximum cluster sizes quantify the average and largest numbers of remnants per cluster, graph diameter indicates the longest shortest path length through a network of clusters, and network modularity is a metric of potential path redundancy that assesses how densely connected remnants are dispersed throughout the network^68^. Modularity assesses clustering of nodes based on linkage density and node location^75^. A network with modularity = 0 has as many links as would be expected in a graph generated with random placement and density of nodes. Positive values indicate greater clustering than expected whereas negative ones are indicative of a diffuse network with many alternative pathways among nodes^75,76^. All network analyses were conducted with package *igraph*^72^ in RStudio.

Because many of the most vagile taxa also have large home ranges or are area-sensitive^77,78^, we examined fragments 3 ha and larger, encompassing the home ranges of a variety of species, including those with larger ranges (e.g. top predators or other megafauna as well as volant species). Although large mammals have large home ranges, they are not restricted to one forest remnant in landscapes that have experienced fragmentation. A network of forest remnants (of varied sizes) can comprise their home range, and smaller remnants can provide connectivity to larger remnants for them. We visualized clusters based on distance between remnants (40–10,000 m).

### Structural connectivity—node-level metrics

In addition to overall-network metrics, we also calculated three node-level metrics to determine which remnants were the most important in supporting connectivity, based on their spatial placement within the network. For each node, we calculated its hub score^72^, which indicated which forest remnants were within dense clusters of other remnants. We identified which nodes were articulation points, which are nodes whereby removal from the network results in fragmenting the network into clusters that require an even larger dispersal distance to coalesce^39,79^. Finally, we calculated the betweenness centrality value of each node, which allowed us to identify forest remnants acting as stepping stones, which are remnants that form the most direct route through the network based on their spatial location^40,80^. Hubs, articulation points, and stepping stones are designations made at coalescence because they indicate nodes that are important in supporting structural connectivity through the whole network. Moreover, the designation as a hub, articulation point, or stepping stone is based purely on a remnant’s spatial location. Connectivity metrics were calculated in package *igraph*^72^ in RStudio. Results were then visualized with ArcGIS 10.7^71^.

## Supplementary Information

Supplementary Information.
