# Supplementary material for: Identifying structural connectivity priorities in eastern Paraguay’s fragmented Atlantic Forest
Source: Sci Rep. 2021 Aug 9;11:16129. doi: 10.1038/s41598-021-95516-3 (PMC8352903; doi:10.1038/s41598-021-95516-3)
Supplement: Supplementary file 1 — Supplementary Information. [file 41598_2021_95516_MOESM1_ESM.docx]

**Identifying structural connectivity priorities in eastern Paraguay’s fragmented Atlantic Forest**

Noé U. de la Sancha^1^, Sarah A. Boyle^2^, and Nancy E. McIntyre^3^

^1^Department of Biological Science, Chicago State University, Chicago, IL USA; delaSancha@msn.com

^2^Department of Biology, Rhodes College, Memphis, TN USA; sarahannboyle@gmail.com

^3^Department of Biological Sciences, Texas Tech University, Lubbock, TX USA; nancy.mcintyre@ttu.edu

*Corresponding Author: delaSancha@msn.com

Supplementary Table 1: Forest loss and fragmentation between 2000 and 2019 in the Atlantic Forest in Paraguay^1^

|  | **Total forest (ha)** | **Total forest (ha) of remnants ≥0.5 ha** | **Total forest (ha) of remnants ≥3 ha** | **# of remnants ≥ 0.5 ha** | **# remnants ≥ 3 ha** | **Mean size (ha) of remnants ≥0.5 ha** | **Mean size (ha) of remnants ≥3 ha** |
| --- | --- | --- | --- | --- | --- | --- | --- |
| 2000 | 3,416,703 | 3,363,364 | 3,268,516 | 113,191 | 35,232 | 29.7 | 92.8 |
| 2019 | 2,403,015 | 2,319,176 | 2,192,306 | 147,770 | 42,081 | 15.7 | 52.1 |
| Change from 2000-2019 | -1,013,688 | -1,044,188 | -1,076,210 | 34,579 | 6,849 | -14.0 | -40.7 |
| **% loss/increase** | **-29.7** | **-31.1** | **-32.9** | **30.6** | **19.4** | **-47.2** | **-43.9** |

^1^Estimates derived from Hansen et al. (2013) forest cover and loss data 2000-2019.

Supplementary Table 2: Summary of Atlantic Forest distribution, loss, and top 200 stepping stones in the 10 departments in 2000 and 2019. First two columns describe the % of forest found in that department from the overall forest cover in the Atlantic Forest of eastern Paraguay at that time.

|  | **Atlantic Forest in Paraguay** | | | **Stepping Stones** | |
| --- | --- | --- | --- | --- | --- |
| **Department** | **% of Atlantic Forest: 2000** | **% of Atlantic Forest: 2019** | **% Change: 2000-2019** | **Number (%): 2000** | **Number (%): 2019** |
| Alto Paraná | 12.1 | 13.4 | -22.0 | 3 (1.5) | 3 (1.5) |
| Amambay | 8.9 | 8.5 | -33.1 | 33 (16.5) | 11 (5.5) |
| Caaguazú | 12.7 | 12.8 | -29.3 | 24 (12.0) | 41 (20.5) |
| Caazapá | 7.7 | 8.6 | -21.3 | 31 (15.5) | 28 (14.0) |
| Canindeyú | 19.4 | 18.1 | -34.1 | 51 (25.5) | 62 (31.0) |
| Concepción | 4.6 | 3.9 | -40.4 | 0 (0.0) | 0 (0.0) |
| Guairá | 3.8 | 4.6 | -14.0 | 12 (6.0) | 19 (9.5) |
| Itapúa | 12.8 | 15.8 | -13.5 | 8 (4.0) | 7 (3.5) |
| Paraguarí | 1.4 | 1.8 | -9.6 | 0 (0.0) | 0 (0.0) |
| San Pedro | 16.6 | 12.5 | -46.9 | 38 (19.0) | 29 (14.5) |

Supplementary Table 3: Number of clusters of forest remnants in eastern Paraguay in 2000 based on different maximum movement distances between patches for networks of different sizes of forest remnants with summary statistics for the clusters identified including the mean number of nodes, median number of nodes, mean cluster size of graph maximum, graph maximum modularity, and mean cluster diameter.

| **Distance traveled** | **PY 2000** | **Mean Nodes** | **Median Nodes** | **Mean Cluster Size** | **Graph Max**  **Modularity** | **Diameter** |
| --- | --- | --- | --- | --- | --- | --- |
| 40 | 35,232 | 1.0 | 1.0 | 1.0 | 0.000000 | 0.0 |
| 100 | 35,228 | 1.0 | 1.0 | 1.0 | -0.125000 | 83.9 |
| 300 | 34,521 | 1.0 | 1.0 | 1.0 | -0.000729 | 771.8 |
| 500 | 29,012 | 1.2 | 1.0 | 1.2 | -0.000110 | 3,709.1 |
| 1000 | 11,388 | 3.1 | 1.0 | 3.1 | -0.000047 | 47,093.1 |
| 2000 | 1,267 | 27.8 | 2.0 | 27.8 | -0.000038 | 344,243.8 |
| 3000 | 212 | 166.2 | 2.0 | 166.2 | -0.000036 | 673,690.1 |
| 4000 | 39 | 903.4 | 2.0 | 903.4 | -0.000035 | 641,804.7 |
| 5000 | 11 | 3,203.0 | 1.0 | 3,202.9 | -0.000035 | 621,705.2 |
| 10000 | 1 | 35,232.0 | 35,232.0 | 35,232.0 | -0.000034 | 604,716.6 |

Supplementary Table 4: Number of clusters of forest remnants in eastern Paraguay in 2019 based on different maximum movement distances between patches for networks of different sizes of forest remnants with summary statistics for the clusters identified including the mean number of nodes, median number of nodes, mean cluster size of graph maximum, graph maximum modularity, and mean cluster diameter.

| **Distance traveled** | **PY 2000** | **Mean Nodes** | **Median Nodes** | **Mean Cluster Size** | **Graph Max**  **Modularity** | **Diameter** |
| --- | --- | --- | --- | --- | --- | --- |
| 40 | 42,079 | 1.0 | 1.0 | 1.0 | -0.250000 | 38.2 |
| 100 | 42,071 | 1.0 | 1.0 | 1.0 | -0.050000 | 97.0 |
| 300 | 41,213 | 1.0 | 1.0 | 1.0 | -0.000600 | 826.6 |
| 500 | 34,561 | 1.2 | 1.0 | 1.2 | -0.000091 | 3,505.2 |
| 1000 | 12,840 | 3.3 | 1.0 | 3.3 | -0.000038 | 45,952.8 |
| 2000 | 962 | 43.7 | 1.0 | 43.7 | -0.000031 | 620,536.1 |
| 3000 | 96 | 438.3 | 1.0 | 438.3 | -0.000029 | 635,471.1 |
| 4000 | 13 | 3237.0 | 1.0 | 3237.0 | -0.000028 | 618,133.6 |
| 5000 | 4 | 10,520.0 | 1.0 | 0.0 | -0.000028 | 608,395.0 |
| 10000 | 2 | 21,040.0 | 21,040.0 | 21,040.5 | -0.000027 | 603,379.2 |
